# Age-Friendly University Models for Engaging With Retired Community Members

**DOI:** 10.1093/geroni/igaa057.1730

**Published:** 2020-12-16

**Authors:** Joann Montepare, Kimberly Farah

## Abstract

The pioneering Age-Friendly University (AFU) initiative, endorsed in 2016 by GSA’s Academy for Gerontology in Higher Education (AGHE), calls for institutions of higher education to respond to shifting demographics and the needs and interests of our aging populations through more age-friendly programs, practices, and partnerships. This symposium will focus on AFU Principle 9 that advocates for engaging actively with an institution’s retired community. Appreciating the diversity of ways in which this principle can be put into practice, participants will discuss the various ways their AFU campuses have built connections with members of their local communities of retired adults. Montepare (Lasell University) will discuss the living and learning model of the institution’s affiliated-retirement community (Lasell Village) that coordinates engagement in 450 credit hours of educational activity as a benefit of residency. Berman (Ithaca College) will discuss the Gerontology Institute’s longstanding relationship with the Longview senior living community and how intergenerational educational and social experiences serve as enriching activities for students, faculty, and residents. Elfenbein (University of North Georgia) will discuss campus collaboration with The Wisdom Project, a community organization of adults 55+ interested in using their skills and talents to benefit their community through action and advocacy projects. Wenrick (Drexel University) will discuss Writers Room, a university-community literary arts program, and their intergenerational co-living initiative, creating a network of homeowners and student tenants whose shared interest in writing and storytelling forms the foundation for meaningful cohabitation.

